# Use of Transversus Abdominis Plane and Intercostal Blocks in Bitches Undergoing Laparoscopic Ovariectomy: A Randomized Controlled Trial

**DOI:** 10.3390/vetsci9110604

**Published:** 2022-10-31

**Authors:** Andrea Paolini, Francesco Santoro, Amanda Bianchi, Francesco Collivignarelli, Massimo Vignoli, Silvia Scialanca, Salvatore Parrillo, Ilaria Falerno, Andrea De Bonis, Martina Rosto, Roberto Tamburro

**Affiliations:** 1 Faculty of Veterinary Medicine, University of Teramo, 64100 Teramo, Italy; 2 Department of Clinical Sciences and Services, The Royal Veterinary College, Hatfield AL9 7TA, UK

**Keywords:** analgesia, dogs, local anesthesia, ropivacaine, ovariectomy, transversus abdominis plane block, intercostal nerve block, systemic analgesia, laparoscopy

## Abstract

**Simple Summary:**

In humans and small animals, the advantages of loco-regional anesthesia include lower peri-operative opioid consumption and less related side effects. Regional techniques which can be used to desensitize the entire abdominal wall in dogs are the transversus abdominis plane (TAP) block, combined with caudal intercostal blocks. The aim of this study was to evaluate whether TAP and intercostal blocks provide analgesia in bitches undergoing a laparoscopic ovariectomy. Twenty client-owned bitches were enrolled in this double-blinded randomized controlled trial, in which the use of the aforementioned techniques was compared with systemic analgesia. Bitches receiving loco-regional anesthesia showed less signs of intra-operative nociception and post-operative pain, with a lower peri-operative opioid requirement. The use of TAP and intercostal blocks seems to be an effective analgesic protocol for bitches undergoing laparoscopic ovariectomy.

**Abstract:**

In humans and dogs, loco-regional anesthesia is associated with lower peri-operative opioid consumption and less related side effects. The combination of transversus abdominis plane (TAP) and intercostal blocks can be used to desensitize the entire abdominal wall in dogs. The aim of this study was to evaluate the effectiveness of TAP and intercostal blocks in bitches undergoing laparoscopic ovariectomy. Twenty client-owned bitches were enrolled in this double-blinded randomized controlled trial. After premedication with dexmedetomidine, methadone and ketamine, the animals were randomized into two groups. Dogs in the TAP group received intercostal blocks from T8 to T10 and a TAP block with ropivacaine. Dogs in the FEN group received a fentanyl bolus and a constant rate infusion for the entire duration of the procedure. Intra-operative cardiovascular stability, post-operative pain scores, rescue opioid requirement, dysphoria during recovery, time to attain sternal recumbency and interest in food at 6 h post-extubation were compared. Bitches in the TAP group received a statistically significant lower amount of rescue fentanyl intra-operatively and methadone post-operatively. Pain scores were lower in the TAP group until 6 h post-extubation. No difference was found for dysphoric recoveries, time to attain sternal recumbency and appetite at 6 h post-extubation. No adverse event was recorded for any of the dogs. The combination of TAP and intercostal blocks can be part of an effective multi-modal analgesic strategy in bitches undergoing laparoscopic ovariectomy.

## 1. Introduction

Surgical pain management is a paramount element of peri-operative care in both humans and animals. Adequate levels of analgesia are often associated with several advantages, such as a quicker recovery and the improvement of recovery quality [1]. Therapeutic options for acute pain include opioids, anti-inflammatory drugs and local anesthetics [2], often administered concurrently in multimodal protocols targeting different phases of pain or nociception. Local anesthetics block the transmembrane neuronal sodium channels, thus interrupting nociceptive transmission to the spinal cord. When compared to systemic analgesia, the use of loco-regional anesthesia has been shown to provide better intra-operative cardio-respiratory stability and lower post-operative pain scores, thereby reducing the overall peri-operative use of opioids and the related side effects [3,4,5]. Moreover, the use of these techniques has been associated with other advantages in people, such as a better quality of recovery, reduced likelihood of central pain sensitization, suppression of the neuro-endocrine stress response, lower immune depression and shorter hospitalization time [5,6].

The transverse abdominis plane (TAP) block entails the ultrasound-guided injection of local anesthetic in the interfascial plane between the internal oblique and the transverse abdominis muscles. Described in both humans and small animals [7,8], this technique aims to desensitize the ipsilateral hemiabdomen (abdominal skin, subcutaneous tissue, mammary glands, abdominal muscles and underlying parietal peritoneum) by blocking the peripheral nerves running within the target plane [9]. The abdominal wall in dogs is innervated by the ventromedial branches of the thoracic nerves from T7 to T13, and the lumbar nerves from L1 to L3 [10]. In dog cadavers, several injection techniques using different volumes have been described, each one resulting in a different spread [11,12,13]. However, with a single injection per side, a complete staining of all the target nerves has never been achieved [8,11,12,14]. In fact, when a single injection is performed in the area between the last rib and the iliac crest (the same approach used in humans), the ventral branches of the spinal nerves from T11 to L3 can be reached by the injectate [8]. Consequently, in order to desensitize the whole hemi-abdomen, the block needs to be extended more cranially. For this purpose, several techniques have recently described, such as an adjunctive sub-costal injection [12] or the use of combined multiple intercostal blocks, up to at least the eighth intercostal nerve [9,14].

In humans and small animals, the TAP block is mainly used to provide intra- and post-operative analgesia in patients undergoing celiotomy or laparoscopic procedures [15,16,17]. 

Although several small animal cadaveric studies, case reports and randomized controlled trials have been published [8,11,12,13,14,18,19,20], to date, no clinical trial has investigated the intra- or post-operative efficacy of the TAP block in dogs undergoing laparoscopic surgery.

The aim of this study was to evaluate the clinical effect of TAP and intercostal blocks on intra-operative nociception and post-operative pain in bitches undergoing a laparoscopic ovariectomy. Our hypothesis reduced nociception and pain, and therefore offers a lower peri-operative opioid requirement in cases receiving the blocks.

## 2. Materials and Methods

This randomized controlled trial was approved by the Scientific Ethics Committee of the University of Teramo, protocol number 24212 with the date 04/10/2021. The owners’ written consent was obtained for all the animals enrolled. Bitches classified as ASA I (American Society of Anesthesiologists) presented to the Veterinary Teaching Hospital “G. Gentile” (University of Teramo) for elective laparoscopic ovariectomy were included in the study. Exclusion criteria were animals younger than 6 months, the presence of any systemic disease, pregnancy, and a body condition score (BCS) <3 or >6. The animals were deemed healthy based on clinical examinations and routine blood tests screenings (hematology and serum biochemistry). The dogs were admitted and hospitalized 24 h before surgery in order to give them the time to acclimatize in their kennels. Food and water were available until 8 h and 30 m before the animals were transferred to the preparation room. Premedication consisted of the intramuscular administration of methadone (0.2 mg kg^−1^, Semfortan; Eurovet Animal Health, Bladel, Netherlands), dexmedetomidine (0.005 mg kg^−1^, Dextroquillan; Fatro, Bologna, Italy) and ketamine (1 mg kg^−1^, Ketavet; MSD Animal Health, Kenilworth, NJ, USA). A 20-gauge intravenous catheter (Jelco; Smiths Medical, Minneapolis, MN, USA) was aseptically inserted into a cephalic vein 15 min later. After 3 min of flow-by pre-oxygenation (150 mL kg^−1^ min^−1^), general anesthesia was induced with propofol (Propovet; Zoetis, Rome, Italy), slowly administered intravenously to effect. Following the endotracheal intubation with a PVC cuffed orotracheal tube of the correct size (Rusch; The Sheridan, Morrisville, NC, USA), the bitches were connected to an anesthetic workstation (Fabius; Dräger, Corsico, Italy) via a circle breathing system, and anesthesia was maintained with isoflurane (Isoflo; Zoetis, Rome, Italy) delivered in 100% oxygen. Immediately after the endotracheal intubation, the animals were connected to a multiparametric monitor (M3046-M2; Philips, Milan, Italy) and the following vital parameters were measured and recorded every 5 min until the end of the procedure: pulse/heart rate; electrocardiogram (ECG); peripheral capillary oxygen saturation (SpO_2_); end-tidal isoflurane concentration (EtISO); end-tidal carbon dioxide (EtCO_2_); respiratory rate; esophageal temperature; and arterial blood pressure, measured invasively (IBP) via a 22-gauge peripheral catheter (Jelco; Smiths Medical, USA) placed in the metatarsal artery. Shaving and the aseptic preparation of the abdomen and caudal bilateral thoracic wall were performed.

The dogs were then randomly assigned to the experimental (TAP) or control group (FEN) using an online randomization tool (www.randomizer.org, accessed on 13 March 2022). 

### 2.1. TAP Group

Ultrasound-guided transversus abdominis plane (TAP) and intercostal blocks were performed bilaterally using approaches previously described in dogs [8]. For the TAP block, a high-frequency (7–12 MHz) 5 cm linear array transducer (LA523; Esaote, Milan, Italy) was positioned longitudinally, parallel to the linea alba, in the area between the posterior margin of the last rib and the anterior margin of the iliac crest. After the correct visualization of all the abdominal muscle layers, a 25-gauge spinal needle (0.5 × 90 mm, Becton; Dickinson, UK) was inserted and advanced in plane, with the ultrasound beam in a cranio-caudal direction and an angle of 20–30° to the skin, until its tip was visualized within the interfascial plane between the internal oblique and the transverse abdominis muscles. After negative aspiration and the use of a small test volume of local anesthetic (0.5 mL) to confirm the correct placement of the needle, 0.5 mL kg^−1^ of ropivacaine 0.2% were injected, and the spread observed in real time under ultrasound guidance.

Subsequently, for the execution of the intercostal blocks, a 5 MHz linear probe was used to identify the intercostal nerves from T8 to T10 in a transverse acoustic window, caudally to each respective rib. A 45 mm quincke (0.5 × 0.45 mm Becton; Dickinson, UK) spinal needle was then introduced in a caudo-cranial direction, and advanced in the plane with the ultrasound beam until its tip was adjacent to the intercostal neurovascular bundle. After negative aspiration, 0.05 mL kg^−1^ of ropivacaine was injected perineurally through. For all the blocks performed, the needle was connected to the syringe containing the local anesthetic (after the removal of the stylet) and primed, in order to avoid ultrasonographic artifacts due to the injection of air bubbles.

For blinding purposes, the dogs in this group received a bolus, followed by a constant rate infusion, of normal saline. The volumes and infusion rates administered corresponded to the ones used for fentanyl in the FEN group.

### 2.2. FEN Group

Dogs in this group received a fentanyl (Fentadon; Eurovet Animal Health, Bladel, The Netherlands) 2 mcg kg^−1^ loading dose, followed by a constant rate infusion (CRI) of 5 mcg kg h^−1^. The same interfascial and intercostal injections described for the TAP group were then performed using an equal volume of normal saline.

All the syringes and infusions were prepared by the same operator in a separate room and labelled in order to be indistinguishable. The anesthetist in charge of administering the treatments was not aware of the content of the syringes.

All dogs were mechanically ventilated using a tidal volume of 10 mL kg^−1^ and a respiratory rate adjusted in order to maintain normocapnia (EtCO_2_ between 35 and 45 mmHg). Ringer’s lactate solution was infused in all dogs at a rate of 5 mL kg^−1^ h^−1^ for the entire duration of the procedure. A periodic assessment of anesthetic depth was based on clinical endpoints (absence of movement, palpebral reflex and jaw tone, and eyes rotated ventrally) and used to adjust the EtISO over time during the procedure. The anesthetic plan was deemed adequate in all dogs before the start of the surgery.

In case of hypotension, once the anesthetic depth was deemed adequate, the anesthetist in charge could decide whether to administer fluids, anticholinergic drugs (atropine) or vasopressors (noradrenaline), based on the clinical evaluation of the case.

Intra-operative rescue analgesia consisted of boluses of 3 mcg kg^−1^ of fentanyl IV (fentanyl (Fentadon; Eurovet Animal Health, Bladel, Netherlands), administered in case of a more than 20% increase in heart rate, systolic, mean and diastolic arterial blood pressure above the baseline values (recorded immediately prior to towel clamps’ placement) or the animal fighting mechanical ventilation. The same anesthetist (AP) performed all the injections/infusions and was in charge of all the cases for the whole duration of the procedure. 

Laparoscopic ovariectomy was performed through three ports (one cranial to the umbilicus and two lateral) as described in a paper published by Case et al. in 2011 [21].

At the end of the surgery, all the enrolled bitches received meloxicam (0.2 mg kg^−1^ IV, Meloxidolor; Le Vet Beheer, Oldeholtpade, Netherlands). The time spent for the execution of the blocks, the duration of surgery and the time between extubation to attain sternal recumbency were recorded for each dog. Post-operative pain was assessed using the short form of the Glasgow pain scale (SF-GCPS) [22] before premedication (baseline), after extubation (T0) and 2, 4, 6 and 24 h after extubation. In case of pain scores ≥ 4/20 (for non-ambulatory dogs) or ≥5/24, a rescue dose of methadone (0.2 mg kg^−1^) (Semfortan; Eurovet Animal Health, Bladel, Netherlands) was administered intravenously. Post-operative dysphoria, interest in food 6 h after extubation and any adverse event or complication were recorded for each dog.

### 2.3. Statistical Analysis

Sample size calculation was performed using a priori analysis based on data previously published by [23]. The difference in post-operative pain scores was chosen as the effect of interest. The sample size effect was estimated to be 0.7, assuming a probability (power) of 0.8 and *α* of 0.05. Data distribution was assessed using the Shapiro–Wilk test. Normally distributed data are presented as mean ± standard deviation, whereas the non-normally distributed values are expressed as median (range).

Fisher’s exact test was used to compare the number of bitches requiring rescue analgesia in the intra- and post-operative period, presence of post-operative dysphoria and the number of animals accepting food 6 h after extubation. The Mann–Whitney U test was used to compare age, weight, time spent for the blocks, surgical time, peak intra-abdominal pressure, median end-tidal isoflurane, total rescue dose of fentanyl and methadone and pain scores at each time point. Statistical analysis was performed using Prism (Version 9.4.1; GraphPad, San Diego, CA, USA). Statistical significance was considered if *p* < 0.05.

## 3. Results

A total of 20 female dogs of various breeds were included in this study and evenly allocated to the two groups. The demographics, pre-operative pain score, time spent to perform the blocks, peak intra-abdominal pressure, baseline hemodynamic values, EtSEVO and duration of surgery and are shown in Table 1.

Age was significantly lower in the TAP group with a median of 12 months (7–24) versus 24 (12–36) in the FEN group (*p* = 0.005). Baseline systolic arterial blood pressure was significantly higher in the FEN group, with a median value of 120 mmHg (108–130), versus 111.5 mmHg (89–129) in the TAP group. No statistically significant difference was found between the groups for weight, time spent for the block, duration of surgery, baseline heart rate and mean arterial blood pressure, median end-tidal isoflurane, peak intra-abdominal pressure and pain score at the time of premedication.

There was a statistically significant difference in terms of total intra-operative rescue fentanyl dose, with 9 mcg kg^−1^ (3–15) in the FEN group and 1.5 mcg kg^−1^ (0–9) in the TAP group (*p* = 0.013), and total dose of rescue post-operative methadone, with 0.2 mg kg^−1^ (0–0.6) in the FEN group and no dose received in the TAP group (*p* < 0.001). Equally, when comparing the number of bitches receiving a rescue treatment intra- and post-operatively, this difference was statistically significant, with 10/10 dogs receiving fentanyl in the FEN group versus 5/10 in the TAP group (*p* = 0.032), and 8/10 dogs requiring methadone in the FEN group versus 0/10 dogs in the TAP group (*p* < 0.001). No statistically significant difference was found when the time from extubation to attain sternal recumbency, number of bitches showing dysphoria and number of dogs eating food at 6 h post-extubation were compared between the groups (Table 2).

**Figure 1:**
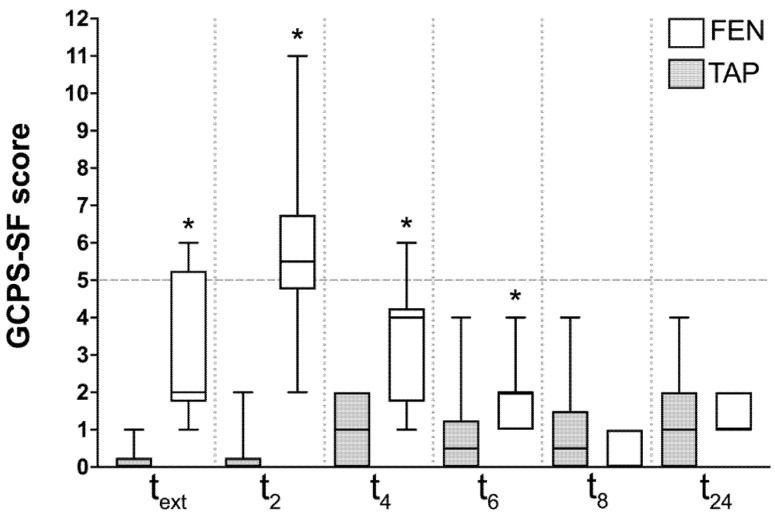
Box and whisker plots of pain scores’ distribution at different time points. The asterisk indicates statistically significant difference between the groups at that specific time point. The dotted line indicates the intervention threshold (score at which rescue analgesia was administered).

The post-operative pain scores differed statistically between the groups from t_ext_ to t6. At t_ext_, the TAP group had a median of 0 (0–1) versus a median of 2 (1–6) in the FEN group; at t_2_, TAP scored 0 (0–2) and FEN scored 5.5 (2–11); at t_4_, TAP scored 1 (0–2) and FEN scored 4 (1–6); and at t_6_, TAP scored 0.5 (0–4) and FEN scored 2 (1–4) (Figure 1).

No adverse event nor complication (including hypotension) was recorded at any time point for any of the animals included.

## 4. Discussion

Based on the findings of this study, this combination of TAP and intercostal blocks was associated with lower peri-operative opioid consumption and a lower post-operative pain scored during the first 6 h post-extubation in a population of healthy bitches undergoing laparoscopic ovariectomy. Similar findings come from other studies in which the TAP block was used as part of a multi-modal analgesia in bitches undergoing ovariohysterectomy [18,19] and queens undergoing ovariectomy via midline celiotomy [23]. The most likely reason for this observation seems to be a desensitization of the abdominal skin, muscles and parietal peritoneum [8,14,24].

More controversial in the literature is the effect of this technique on visceral pain [25,26]. In light of the results of this trial, the blocks performed may have resulted in some degree of visceral analgesia in the studied population, although this can only remain purely speculative. In fact, in laparoscopic procedures, the insufflation of the peritoneal cavity with carbon dioxide (a volatile acid) is well recognized as an important nociceptive and painful stimulus in people [27,28,29], together with the somatosensory stimulus resulting from the distension and surgical trauma of the abdominal wall. In small animals and humans, one of the advantages of laparoscopic surgery is a lower level of peri-operative pain [30,31,32]. However, visceral pain in dogs is still poorly recognized and scored, and it is therefore more challenging to exactly quantify the net analgesic benefit of laparoscopic surgery in this species [30]. One limitation of this study is that its design does not yield any firm conclusion about the effect of the used techniques on visceral pain.

Another main bias of the present study is the difference in co-interventions between groups, represented by the fentanyl infusion only being administered in the FEN group. When designing this study, the absence of any intra-operative systemic analgesia in the control group was deemed ethically debatable, and fentanyl was chosen for this purpose.

The main consequence of a similar design could be a significant difference in baseline cardio-vascular parameters between groups, resulting in a lower intra-operative absolute interventional threshold in the group of dogs receiving fentanyl, hence a higher likelihood of receiving rescue analgesia. However, when comparing pre-incisional hemodynamic values between groups, the only parameter resulting in a statistically significant difference was systolic arterial blood pressure, which was unexpectedly higher in the TAP group. For this reason, notwithstanding the inherent bias, it seems unlikely that this had a profound impact on the results.

Based on the complete absence of a post-operative rescue analgesia requirement in bitches receiving the blocks, and the statistically significant difference between the groups, there seems to be an important effect on the loco-regional techniques in this phase, up to 6 h post-extubation. Nevertheless, during recovery, all the dogs received meloxicam, which in previous studies proved to be effective as the sole post-operative analgesic treatment in bitches undergoing ovariectomy [32]. The difference in pain scores at 6 h does not yield a clear explanation for the maximum duration of action of local ropivacaine, which is described to be shorter [33]. Some other factors could therefore play a role, such as a physiological pain reduction over time and the return to the pre-operative pain scores within a few hours, a phenomenon already reported after ovariohysterectomy in bitches, regardless of the analgesic protocol used [34].

The short form of the Glasgow composite pain scale is a validated tool in dogs, although the behavioral component unrelated to pain may affect its diagnostic power [22].

A reduction in peri-operative opioid requirements in the bitches receiving the blocks can be considered one of the main findings of this study. Despite being the mainstay of systemic analgesia, opioids are associated with several systemic side effects, such as nausea, vomiting and gastro-intestinal dysmotility, sedation or dysphoria and transient immune depression in humans and small animals [35,36]. Even though the overall clinical impact of these side effects is still not completely quantified in dogs, the reduction in the total opioid dose can be considered a clinical advantage, potentially contributing to a better recovery quality, lower morbidity and shorter hospitalization time, as demonstrated in people [36].

When demographic data were compared, age turned out to be statistically lower in the TAP group compared to the FEN group. Although it is impossible to exclude some effect of this difference on the outcomes of interest, as the vast majority of subjects are young adults, this can be considered a minimal bias.

A cadaveric study by Bruggink and colleagues [37] showed a direct proportionality between the volume injected and the number of nerves stained when the TAP block with a single lateral injection was performed. In this paper, the authors found that a mean number of approximately four nerves were stained when a volume of 1 mL per kg was injected. Notwithstanding these results, a lower volume was chosen for this study, mainly to avoid the use of a high total dose of local anesthetic or an inadequate concentration due to dilution.

All loco-regional techniques have some inherent risk of complication. In humans, the inadvertent puncture of abdominal organs (mainly liver, spleen and gastro-intestinal tract) has been reported, although it appears to be a rare event [38]. The results of this study are in agreement with a recently published retrospective study in which no complications were associated with the TAP block in a canine population undergoing ovariohysterectomy [19]. When intercostal blocks are performed, each needle insertion may cause a lung puncture or the intramuscular, intravascular, and interpleural injection of local anesthetic as the main complications. Despite the current lack of clinical data in small animals, it is reasonable to think that the rate of adverse events is decreased when these techniques are performed under ultrasound guidance.

In this study, the TAP was performed with a single injection as described by Schroeder and colleagues, who reported a staining of T11 in only 20% and L3 in 30% of the cadavers [8]. A similar approach in cats resulted in a similar pattern, with L2 and L3 stained in 2/8 cadavers, and L2 in 1/8. [39]. Based on the aforementioned results, it would be reasonable to think that the single injection technique would result in a partial block. Interestingly, however, in clinical studies and case reports, this approach (or similar) provided satisfactory analgesia in dogs and cats. [23,40]. One reason for this discrepancy could be that cadaveric nerve staining can be a poor predictor of the local anesthetic spread in vivo. In fact, studies on single-shot multi-segmental loco-regional blocks in humans show how the desensitized region extends over the area stained with methylene blue or iodine, in contrast to both cadaveric models and in vivo [41]. Nevertheless, considering the evidence about a two-point injection technique resulting in a more extended and consistent spread in cadavers, the latter can be considered a safer approach. [12,39]

The combination of the TAP and intercostal blocks is not the only described option to extend the desensitization cranial to T11. In fact, [12] recently described a modified TAP approach with the adjunct of a sub-costal injection, resulting in a similar cranial extension of the block in a cadaveric model [11]. Considering the time needed to extend the clipped area cranially, to find the correct acoustic window and to perform multiple intercostal injections (with the associated potential complications, such as intravascular injections and pleural punctures [42]), the subcostal injection could be an easier, quicker and safer option, although no clinical data are currently available for this approach.

Another limitation of this study is the small sample size, although this resulted from a power calculation. Furthermore, for some secondary outcomes of this study, the possibility of a type II error caused by insufficient power should be considered, as the difference in post-operative pain scores was used as the only considered biological effect when performing the power calculation.

## 5. Conclusions

The use of the TAP block as described by Schroeder and colleagues, associated with intercostal blocks from T8 to T10, can be a valid part of a multi-modal analgesic protocol in bitches undergoing laparoscopic ovariectomy, thereby reducing the peri-operative opioid requirement. Further studies are needed to confirm this finding in other canine populations.

None of the bitches in this population experienced any adverse side effects at any time, although further studies are needed to investigate the safety of the techniques used.

## Figures and Tables

**Table 1:** Demographic data, time spent for the block, peak abdominal pressure, baseline hemodynamic values, median intra-operative EtISO and duration of surgery. Values expressed as median (range).

	TAP	FEN	*p* Value
Age (months)	12 (7–24)	24 (12–36)	0.005
Weight (kg)	21.3 (15–36)	30 (20–39)	0.077
Pain score at the time of premedication	0 (0–3)	0 (0–2)	0.981
Time spent for the block (min)	25 (13–34)	26 (15–36)	0.867
Peak intra-abdominal pressure (mmHg)	8.5 (8–10)	9 (8–12)	0.633
Baseline heart rate (bpm)	66 (45–90)	66.5 (44–70)	0.381
Baseline SAP (mmHg)	111.5 (89–129)	120 (108–130)	0.027
Baseline MAP (mmHg)	75 (67–86)	79.5 (69–96)	0.085
Median intra-operative EtISO (%)	1.2 (1.1–1.3)	1.2 (1.15–1.2)	0.696
Duration of surgery (min)	82.5 (50–120)	87 (75–195)	0.446

SAP: systolic arterial blood pressure; MAP: mean arterial blood pressure.

**Table 2:** Number of dogs requiring rescue fentanyl and methadone, showing post-operative dysphoria and eating food 6 h post-extubation: values expressed as number of cases on total number of dogs per group. Total dose of intra-operative rescue fentanyl, post-operative methadone and time to attain sternal recumbency: values expressed as median (range).

	TAP	FEN	*p* Value
Dogs requiring rescue fentanyl	5/10	10/10	0.032
Total fentanyl dose (mcg kg^−1^)	1.5 (0–9)	9 (3–15)	0.013
Dogs requiring rescue methadone	0/10	8/10	<0.001
Total methadone dose (mg kg^−1^)	0	0.2 (0–0.6)	<0.001
Time to sternal recumbency (min)	30 (19–80)	45 (16–70)	0.589
Dogs showing dysphoria	0/10	3/10	0.21
Dogs accepting food at 6 h	8/10	4/10	0.169

## Data Availability

The data generated in this study is already added in the tables of this article. If you need any further information, please feel free to contact authors.
